# Anesthesia for subglottic stenosis in pediatrics

**DOI:** 10.4103/1658-354X.57882

**Published:** 2009

**Authors:** Essam A. Eid

**Affiliations:** *Department of Anesthesia Liver Institute, Menoufia University, Egypt, Assoc Professor, Department of Anesthesia, College of Medicine, King Saud University, Riyadh, Kingdom of Saudi Arabia*

**Keywords:** *Anesthesia*, *subglottic stenosis*, *pediatrics*

## Abstract

Any site in the upper airway can get obstructed and cause noisy breathing as well as dyspnea. These include nasal causes such as choanal atresia or nasal stenosis; pharyngeal causes including lingual thyroid; laryngeal causes such as laryngomalacia; tracheobronchial causes such as tracheal stenosis; and subglottic stenosis. Lesions in the oropharynx may cause stertor, while lesions in the laryngotracheal tree will cause stridor. Subglottic stenosis is the third leading cause of congenital stridors in the neonate. Subglottic Stenosis presents challenges to the anesthesiologist. Therefore, It is imperative to perform a detailed history, physical examination, and characterization of the extent and severity of stenosis. Rigid endoscopy is essential for the preoperative planning of any of the surgical procedures that can be used for correction. Choice of operation is dependent on the surgeon's comfort, postoperative capabilities, and severity of disease. For high-grade stenosis, single-stage laryngotracheal resection or cricotracheal resection are the best options. It has to be borne in mind that the goal of surgery is to allow for an adequate airway for normal activity without the need for tracheostomy. Anesthesia for airway surgery could be conducted safely with either sevofluraneor propofol-based total intravenous anesthesia.

## INTRODUCTION

Any site in the upper airway can get obstructed and cause noisy breathing as well as dyspnea. These include nasal causes such as choanal atresia or nasal stenosis; pharyngeal causes including lingual thyroid; laryngeal causes such as laryngomalacia; tracheobronchial causes such as tracheal stenosis; and subglottic stenosis. Lesions in the oropharynx may cause stertor, while lesions in the laryngotracheal tree will cause stridor. Subglottic stenosis is the third leading cause of congenital stridors in the neonate. Most cases are self-limited, but some need interventions that require a multidisciplinary approach.[1]

In the early twentieth century, the incidence of subglottic stenosis (SGS) in infants was rare. In the 1960s the incidence of acquired SGS began to dramatically increase in this population. This resulted from the increased survival of low-birth-weight babies and the increased use of intubations. Long-term intubations became accepted as an alternative to tracheotomy.[2] Premature infants tolerate prolonged intubations better than adults, most probably due to more yielding and pliable airway cartilages. Tracheotomy should be considered after 50 days of intubations in neonates as recommended by Lesperance and Zalzal.[3]

## ANATOMY

The subglottic area is defined as the area extending from the lower surface of the true vocal cords to the lower surface of the cricoid cartilage. In adults this corresponds to approximately 10 mm lower than the anterior commissure and 5 mm lower than the posterior commissure.[4]

The infant larynx differs significantly in size and position when compared to the adult larynx. At birth, the infant larynx is approximately one-third the size of the adult larynx, however, the infant larynx is proportionately larger than the adult larynx compared to the remainder of the tracheobronchial system. The vocal process of the arytenoid takes up half the length of the vocal cord in the infant larynx, while it only takes up about a quarter of the length of the vocal cord in the adult. The narrowest portion of the airway in an older child or adult is the glottic aperture, while the narrowest part of the airway in an infant is the subglottis. The subglottis in infants measures approximately 4.5 by 7 mm. A diameter of 4.0 mm is considered the lower limit of normal in a full-term infant and 3.5 mm in a premature infant. One millimeter of edema circumferentially, in the subglottis, reduces the cross-sectional area by 60%. The infant larynx is positioned higher in the neck than the adult larynx. The superior border of the larynx of the infant is located at about the level of the first cervical vertebra, with the cricoid positioned at about the fourth cervical vertebra. In comparison, the adult cricoid rests at about the level of the sixth cervical vertebra. The structures of the infant larynx are more pliable and less fibrous, making the infant airway more susceptible to narrowing from edema and less easily palpable.[5]

### Etiology

Ninety-five percent of the cases of acquired SGS in infants results from prolonged endotracheal intubations.[6] The reported incidence of subglottic stenosis in intubated infants ranges from 1-8%, but if very low birth weight infants (<1500 gm) are excluded the incidence is less than 0.1%.[5] The pathogenesis of acquired subglottic stenosis is mainly due to mucosal compression by the endotracheal tube, leading to mucosal edema, ischemia, and ulceration. This ulceration leads to perichondritis and chondritis. Healing of the affected cartilage is by secondary intention and deposition of fibrous tissue. The cartilage then shows necrosis and collapses. The final result of this process is a weak cartilage framework and a firm scar narrowing the subglottic airway. Healing of the subglottic ulceration, secondary to prolonged endotracheal intubation, is found to be quite similar to the healing events in skin and suggest that SGS is the mucosal equivalent of a ‘keloid’, or perhaps, more appropriately, a ‘hypertrophy scar’.[7] Risk factors for the development of acquired subglottic stenosis in neonates, besides prolonged endotracheal intubation, include, the size of the endotracheal tube, increased motion of the endotracheal tube, repeated or traumatic intubations, birth weight less than 1500 gm, upper respiratory tract infection, compromised immune status, presence of nasogastric tubes, and the presence of Pathologicalal gastroesophageal reflux (GER).[6,7]

Gastroesophageal reflux has been proposed as a medical condition that may exacerbate the pathogenesis of SGS, may cause re-stenosis after LTR (Laryngotracheal Reconstruction) repair, and may be the sole cause of stenosis in infants with no previous history of endotracheal intubation.[8] Pathological GER, that is, GER with tracheal aspiration of gastric contents, has been recognized as a single episode of an esophageal pH less than 4.0 for more than 10% of the recording time. GER could be diagnosed by the interpretation of data collected from a magnified high kilovoltage tomogram of the airway (MAG airway), Barium video-esophagram, gastroesophageal reflux scintiscan (GER scan), or by the very recent finding of a direct bronchoscope (edema and erythema of the arytenoids and trachea, loss of outline of the tracheal rings, and blunting of the carina), with the respiratory status of the infant.[9] More than 80% of the infants with subglottic stenosis are seen to have pathological GER. Routine use of anti-reflux therapy for all cases of SGS have been prescribed as an essential step in the management of those children.[9] Despite the lack of definitive evidence for a cause and effect relationship between GER and SGS, Cotton and O'Connor[10] stated that, a “reflux workup” is considered essential for the success of LTR. Moreover, empirical treatment of GER, that is, metoclopramide, ranitidine, and omeprazole, has been recommended by Burton *et al*.[7] for all infants undergoing LTR, even if they do not have symptoms of GER.

### Diagnosis

**Figure 1:**
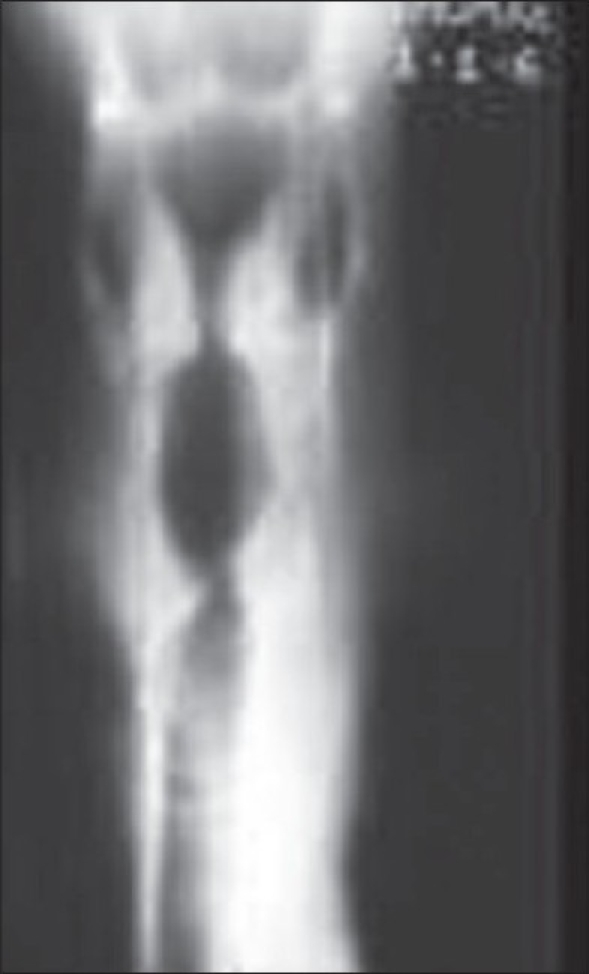
Plain X-ray

When these children present, it is important to perform a thorough history and physical examination. It is important to note any birth injury or intubation, as well as prematurity. The timing, onset, and duration of stridor, voice/cry quality, feeding abnormalities or failure to thrive, cyanosis, and possible foreign body aspiration are important to document, as also, recurrent croup or hospitalizations for respiratory illnesses. The physical examination should include a thorough head and neck examination, as well as careful characterization of the stridor and signs of respiratory distress. A flexible laryngoscopic examination should also be performed. At that time, an assessment of laryngomalacia, vocal cord paralysis, laryngopharyngeal reflux, or other laryngeal pathology can be elicited. Plain X-ray for the neck and upper chest might reveal the stenotic segment [Figure 1].[10]

The gold standard for the diagnosis of any laryngotracheal abnormalities is direct laryngoscopy and tracheobronchoscopy under general anesthesia. This should be performed in the operating room, with an experienced anesthesiologist. It is important to delay endoscopy for at least two weeks following an acute episode of croup, to minimize the risk of postoperative airway obstruction. The potential need for tracheotomy should be discussed with the patient's family prior to endoscopy. A rigid bronchoscope or a rod lens telescope may be used to assess the airway. The important things to document during endoscopy are as follows: (1) the outer diameter of the largest bronchoscope or endotracheal tube that can be passed through the stenotic segment, (2) the location/subsites (glottis, subglottis, trachea) and length of the stenosis, (3) other separate sites of stenosis, (4) other airway anomalies in infants (clefts, webs, cricoarytenoid joint fixation, neoplasms, etc.), and (5) reflux changes. After removing the sizing endotracheal tube or bronchoscope it is important to observe the stenotic segment for edema, which may result in the need for tracheostomy.[11]

**Figure 2:**
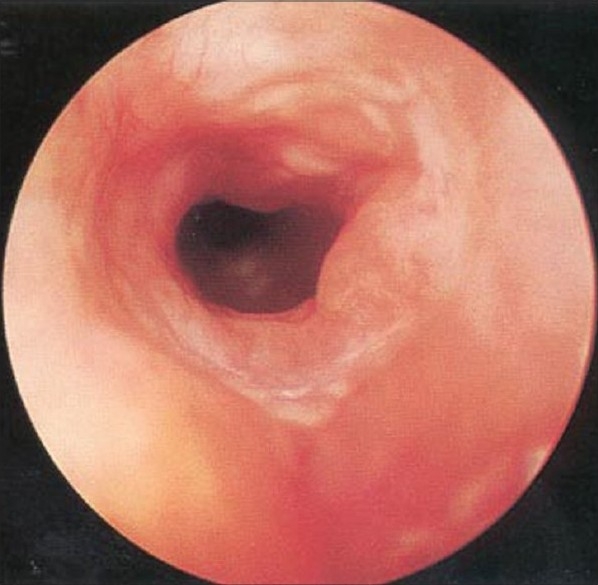
Myer-Cotton grade I

**Figure 3:**
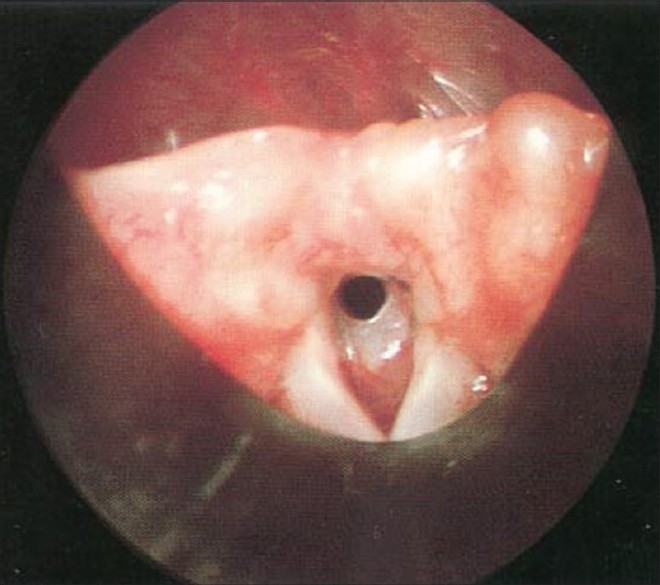
Myer-Cotton grade II

**Figure 4:**
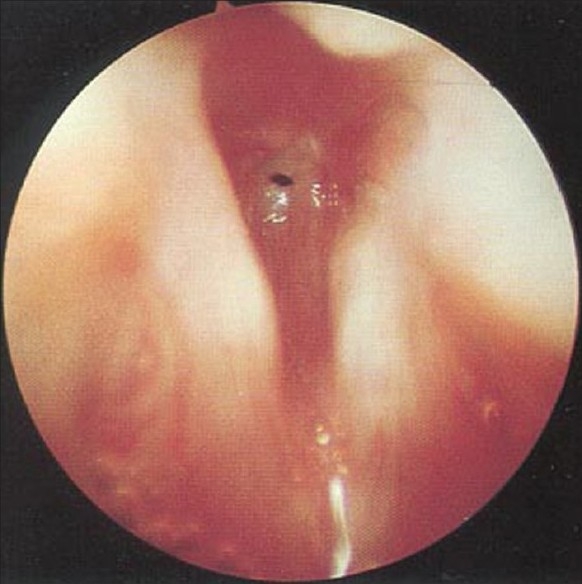
Myer-Cotton grade III

**Figure 5:**
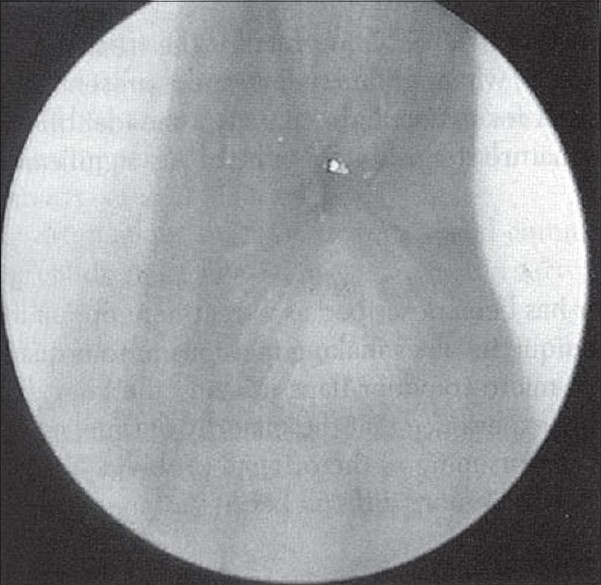
Myer-Cotton grade IV

There are two widely excepted staging systems for classifying subglottic stenosis: The Myer-Cotton grading system and the McCaffrey system.[12] The Myer-Cotton staging system is useful for mature, firm, circumferential stenosis, confined to the subglottis. It describes the stenosis based on the percent relative reduction in the cross-sectional area of the subglottis, which is determined by endotracheal tubes of differing size. Four grades of stenosis are described in this system: Grade I lesions have less than 50% obstruction, grade II lesions have 51 to 70% obstruction, grade III lesions have 71 to 99% obstruction, and grade IV lesions have no detectable lumen or complete stenosis [Table 1, Figures 2–5]. The McCaffrey system classifies laryngotracheal stenosis on the basis of the subsites involved and the length of the stenosis. Four stages are described: Stage I lesions are confined to the subglottis or trachea and are less than 1 cm long, stage II lesions are isolated to the subglottis and are greater than 1 cm in length, stage III are subglottic/tracheal lesions not involving the glottis, and stage IV lesions involve the glottis [Table 2].

**Table 1:** Myer-Cotton grading score[12]

Classification	From	To
Grade 1	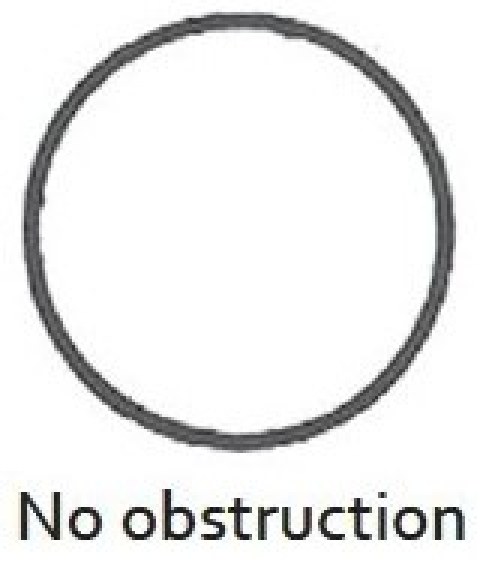	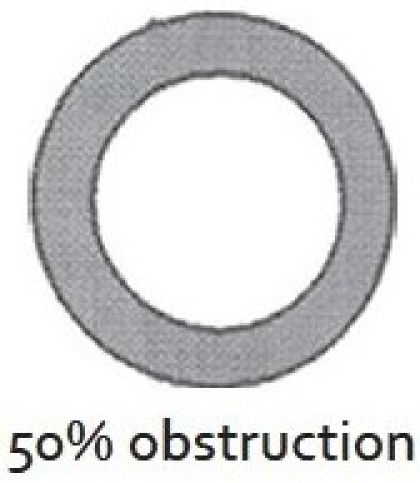
Grade II	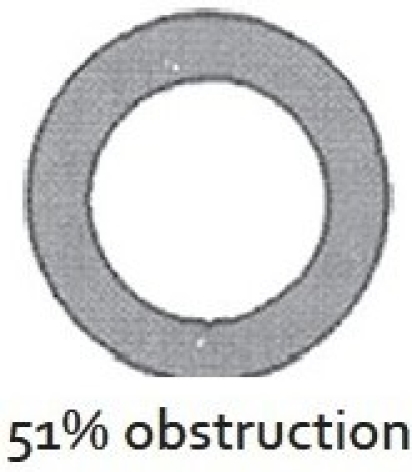	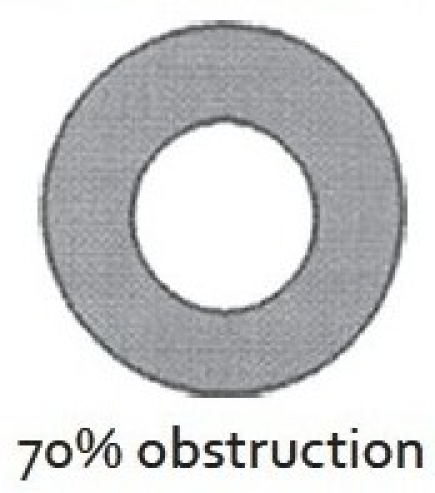
Grade III	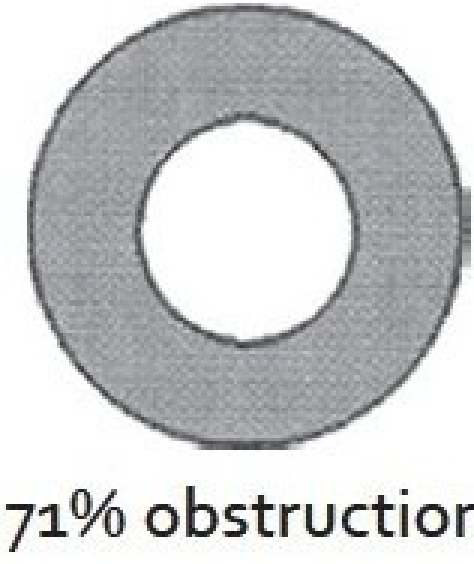	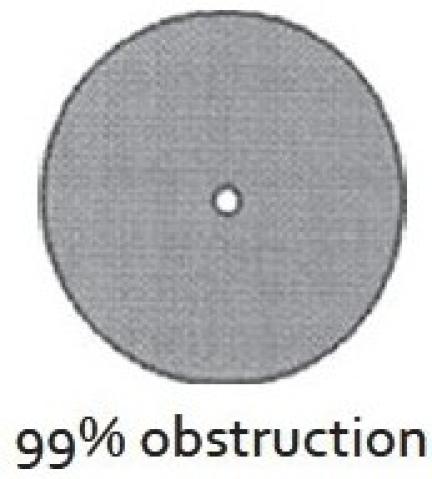
Grade IV	No detectable lumen	

**Table 2:** The McCaffrey system

Stage 1	Stage 2	Stage 3	Stage 4
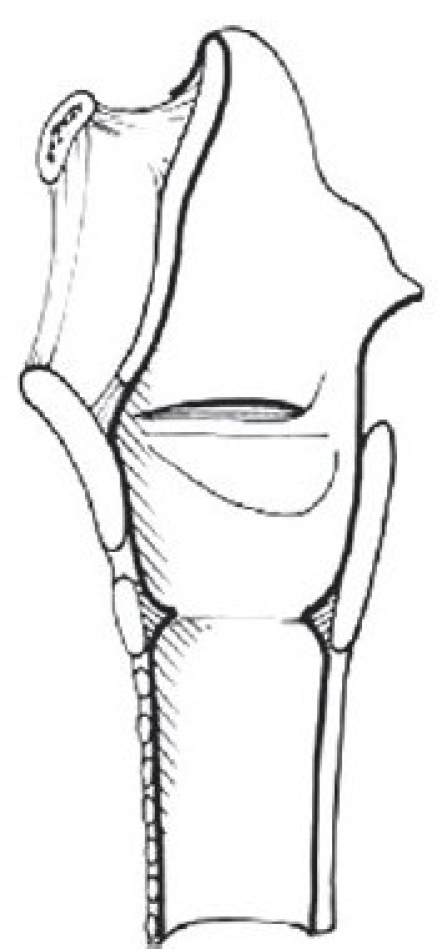	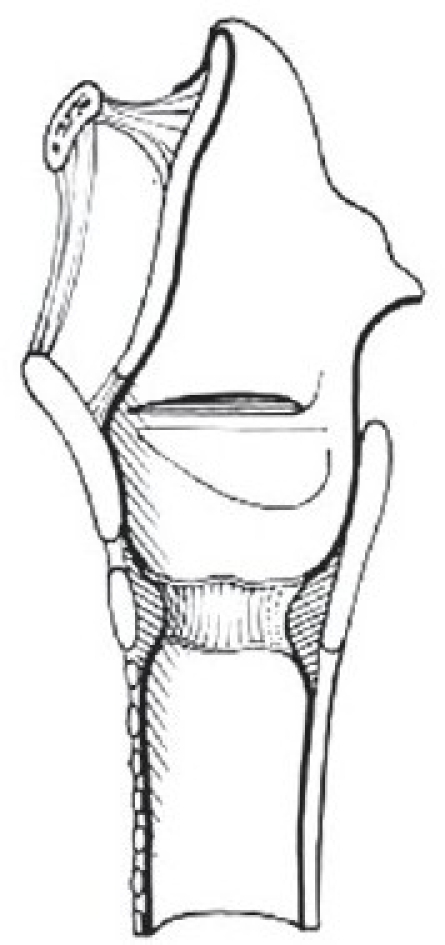	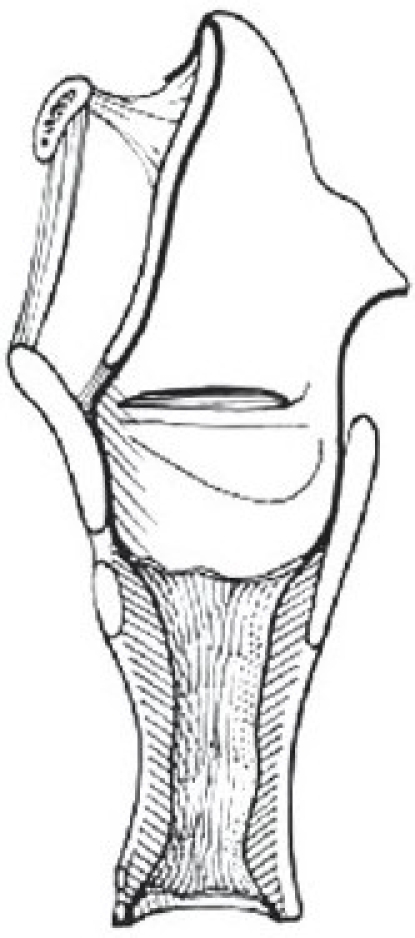	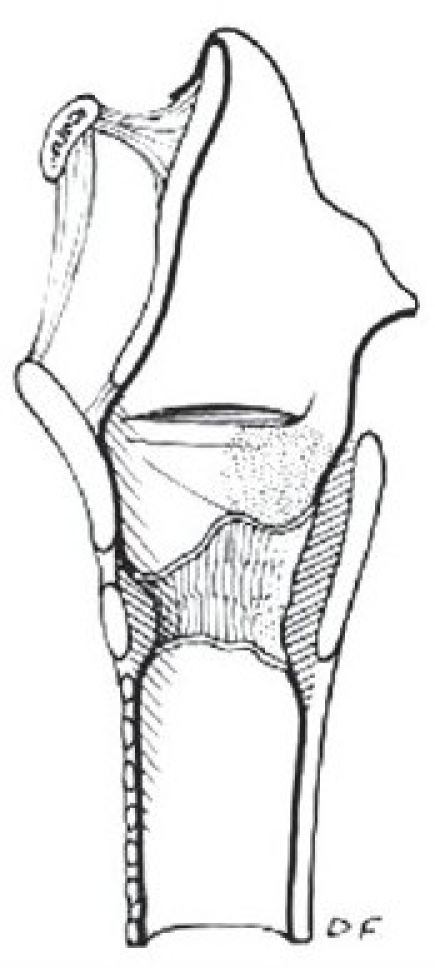

### Surgical management

The management of SGS ranges from observation with supportive care in times of exacerbations, to complicated surgical reconstructions of the patient's airway. Most grade I and II lesions are managed with observation (outpatient fiberoptic nasopharyngoscopy, bronchodilators, and antireflux medications). If a grade II lesion becomes symptomatic, causing a decrease in exercise tolerance or respiratory distress, then endoscopic repair, dilation, or an expansion procedure can be undertaken. CO_2_ laser has also been used for lesions that are thin and circumferential. However, failure to laser ablation tends to occur with thick, circumferential cicatricial scarring, greater than 1 cm in vertical dimension and posterior commissure involvement. Complications of laser include chondritis and perichondritis, as well as re-stenosis. High grade stenosis, grades III and IV, will need an open surgical procedure.[13] The goal of surgery is to have the patients with adequate airway to allow for normal activity without the need for tracheostomy. The secondary goal is to have a single-stage procedure, minimal postoperative morbidity, and minimal hospital stay. Most surgeries are performed in spring and summer, when the chance of developing respiratory system viral infection is lower. However, postoperative complications are common among those infants. These include bleeding, pneumothorax, pneumomediastinum, recurrent laryngeal nerve injury, slipped graft, slipped stent, plugged stent, wound infection, keloid formation, suprastomal/infrastomal collapse, re-stenosis, tracheocutaneous fistula, granulation tissue, and death.

### Anesthetic management

Anesthetic management of infants with SGS is challenging. Infants with SGS are always sick with major comorbidities. Coping with central airway obstruction and sharing the airway with the surgeon makes it easily and critically compromised. Total airway obstruction is a possibility that has to be faced occasionally.[14] Those infants are almost always present with a co-pathology related to central airway obstruction, which includes pulmonary sepsis, obstructive airway disease, and prematurity.[5] Infants with SGS cannot be categorized as ASA I or II because of all of these comorbidities. Most of the infants with SGS embody a category of airway problems for which there is no fail-safe procedure: The pathology is sufficiently central that transtracheal ventilation, tracheostomy, or a similar surgical airway would not relieve the obstruction.[11] Coughing for example is likely to set a chain of events that can reduce the airway to a critical size and even total obstruction. For these two reasons, anesthetic management plans for the airway are not modeled on those traditionally advocated for non-airway surgery or even other airway procedures.

A significant upper airway problem and subglottic stenosis may present in the same patient (cleft lip/palate, tracheomalacia, upper respiratory tract infection). Therefore, it is possible that local anesthesia approaches for securing airways and gaseous inductions might induce complete airway obstruction. Moreover, the risk of precipitating a life-threatening coughing fit with gaseous induction is very high, therefore, inhalational induction was contraindicated. This advice could be qualified in the light of the introduction of sevoflurane into clinical practice.[15] It is now possible to induce anesthesia without sufficient irritation of the airways to provoke coughing, but in this patient population, the margin for safety is small and there are no suitable surgical airways to deal with the consequent total airway obstruction. Total intravenous anesthesia technique (TIVA) for these sick cases — that of inserting rigid instrumentation and any subsequent hemodynamic pressor response — is ablated by using the modern intravenous agents. In principle, the technique has altered little over the years, except for the use of more specific pharmacological agents, such as, propofol, remifentanil, and dexmedotomedine, when they became available. Thus, propofol replaced etomidate; and remifentanil infusions replaced alfentanil, which in turn had replaced fentanyl.[15–17] A multidisciplinary approach is taken to manage this complex problem and consultation with the pediatrician, anesthetist, and surgeon is essential. Anesthesia techniques for endoscopic surgery for SGS should fulfill the requirements of anesthesia and surgery for the upper airway. It should provide the surgeon with a clear, immobile field, and a sufficient area to work in, and the anesthesiologist with minimal homodynamic changes, which are characteristic to airway manipulation, to protect the trachea and to ensure ventilation and oxygenation.[18]

There are two anesthetic techniques that are widely used: I - TIVA technique, where the infant receives propofol in a stepped fashion of 80 ug/kg/min to reach an infusion rate of 240 ug/kg/min in 10 minutes, using a syringe pump. At this point we are able to ventilate the lungs with relative ease (Han grade 1: Ventilation by mask without adjuncts) and the patient is unresponsive. The infusion rates of propofol encompass the dose range used clinically for pediatric patients undergoing ambulatory procedures.[19] Increments of propofol such as 0.5 mg/kg, are used to maintain the anesthesia level. II - Inhalational (Sevoflurane) technique, where anesthesia is induced (pupils become central and infants are easy to ventilate) and maintained with sevoflurane.

Once the induction criteria of anesthesia have been fulfilled with the above techniques, one puff of lidocaine 10% is applied to the hypopharynx and another one to the vocal cords. Ventilation is conducted initially by hand, with a Mapleson C system. Depth of anesthesia is maintained by boluses of propofol or sevoflurane, to keep the heart rate and blood pressure within ±20% of the basal line. Muscle relaxants are not used and if the patient resumes spontaneous breathing, the anesthesia level will be deepened with either propofol or sevoflurane, accordingly. Lung ventilation is achieved with the ventilating bronchoscope. Heart rate, mean blood pressure, and oxygen saturation must be monitored continuously. At all times, the tracheotomy set must be available. Once apnea is achieved and pupils become central, the surgeon is allowed to introduce the rigid bronchoscope. A purpose-built tube connector is attached to the ventilation port of the bronchoscope and to the gas outlet. Lungs must be ventilated with O_2_ 40% in air, in cases of prematurity and laser ablation. Manual ventilation is performed to provide appropriate tidal ventilation. Lungs are ventilated with a face mask, in between the withdrawal intervals of the bronchoscope. The airway having been secured and maintained, the conduit is then free for the introduction of equipment, for end-tidal carbon dioxide monitoring. The anesthesia circuit is switched to be driven from an air source during laser ablation.

**Figure 6:**
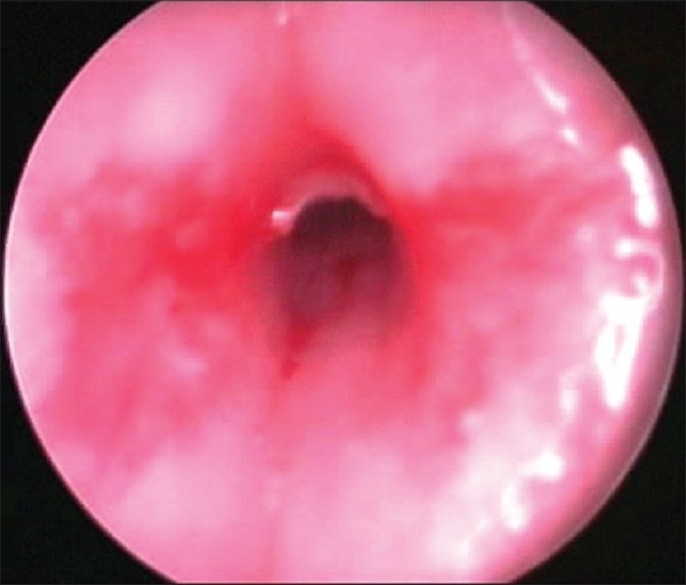
Subglottic edema

**Figure 7:**
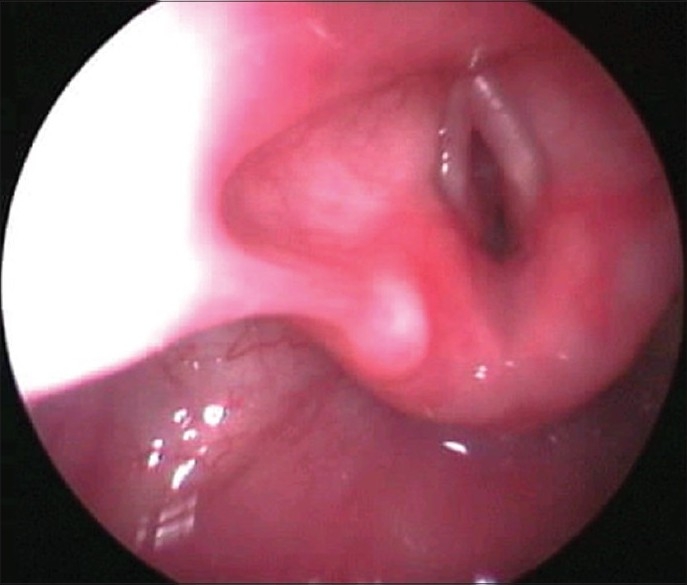
Glottic edema

Before starting surgical manipulation, infants received IV dexamethaszone 0.3 mg/kg, and were exposed to GER assessment by recording the endoscopic signs of reflux (edema and erythema of the arytenoids and trachea, loss of outline of the tracheal rings, and blunting of the carina) and the pH changes were recorded by a four-channel (Castell)-type solid-state manometer catheter, with esophageal motility analysis software for lower and upper esophageal sphincter dual-sensor pH catheters (Medtronic Functional Diagnostics, Shoreview, Minn). At the end of surgery, the infants were intubated with an endotracheal tube (ETT) that permitted air leakage at 20 cm H_2_O and shifted to the neonatal intensive care unit (NICU). Extubation was performed after 24 hours of mechanical ventilation. Cases with obvious laryngeal and subglottic edema [Figures 6 and 7] should be ventilated for 48 hours to provide time for the edema to resolve. Tracheal extubation for all cases should be done in the Operating Room (OR) theater, where all the facilities for emergency and tracheostomy are available.[20]

Finally, the anesthetist must remember that more than 95% of acquired subglottic stenosis in infants and neonates is attributed to a previous endotracheal intubation. There is no “safe” period of intubation, as subglottic stenosis has been reported in as early as 17 hours of intubation.[21] An endotracheal tube should allow an air leak at an inspiratory pressure of 20 cm H_2_O. The absence of an audible air leak is indicative of an excessively large tube.[22] A study done in France reported a very low incidence of SGS in neonates and premature neonates intubated for more than four weeks with reasonable-sized tubes. The authors recommend the following tube sizes based on body weight: Infants <2500 g size 2.5 mm, children 2500-4000 g size 3.0 mm, children >4000 g size 3.5 mm.[23]

Airway surgery has typically necessitated a deeper level of anesthesia for control of airway reflexes and the fluctuations in the hemodynamic parameters, which are characteristic of this surgery. Second, the anesthesia technique must not contain opioids, however, the use of a short acting opioid (remifentanil) after securing the airway is now recommended and constitutes an important element in saving anesthesia. Third, the anesthesia technique should provide a smooth and slow onset of sedation, and it must be with minimal respiratory depression, good control of airway reflexes, and stable hemodynamic profile.[24]

## POSTOPERATIVE CARE

Patients should be admitted to the intensive care unit (ICU), and care must be coordinated among the ICU team and the Pediatrics and Otolaryngology Departments. The patients require sedation, but the length of sedation will vary on the age of the child, and the procedure performed. In children over four years of age, there is a better chance of weaning sedation within 48 hours after the procedure. Aggressive and meticulous tracheostomy care and pulmonary toilet needs are to be undertaken. Postoperative antibiotics, antireflux medications, and dexamethasone are also indicated.[25]

## CONCLUSIONS

It is imperative to perform a detailed history, physical examination, and characterization of the extent and severity of stenosis. Rigid endoscopy is essential for the preoperative planning of any of the surgical procedures that can be used for correction. Choice of operation is dependent on the surgeon's comfort, postoperative capabilities, and severity of disease. For high-grade stenosis, single-stage laryngotracheal resection or cricotracheal resection are the best options. It has to be borne in mind that the goal of surgery is to allow for an adequate airway for normal activity without the need for tracheostomy. Anesthesia for airway surgery could be conducted safely with either sevoflurane- or propofol-based total intravenous anesthesia.
